# Novel low-nitrogen stress-responsive long non-coding RNAs (lncRNA) in barley landrace B968 (Liuzhutouzidamai) at seedling stage

**DOI:** 10.1186/s12870-020-02350-2

**Published:** 2020-04-06

**Authors:** Zhiwei Chen, Qi Jiang, Panpan Jiang, Wan Zhang, Jianhua Huang, Chenghong Liu, Nigel G. Halford, Ruiju Lu

**Affiliations:** 1 Biotechnology Research Institute, Shanghai Academy of Agricultural Sciences, Shanghai, 201106 China; 2 Shanghai Key Laboratory of Agricultural Genetics and Breeding, Shanghai, 201106 China; 3 College of Fisheries and Life Science, Shanghai Ocean University, Shanghai, 201306 China; 4 Shenzhen RealOm ics (Biotech) Co., Ltd., Shenzhen, 518081 China; 5 Suzhou Polytechnic Institute of Agriculture, Suzhou, 215008 Jiangsu China; 6 Plant Sciences Department, Rothamsted Research, Harpenden, Hertfordshire, AL5 2JQ UK

**Keywords:** Long non-coding RNAs, Barley, *Hordeum vulgare*, Low-nitrogen stress, Nitrogen use efficiency, RNA-seq

## Abstract

**Background:**

Reducing the dependence of crop production on chemical fertilizer with its associated costs, carbon footprint and other environmental problems is a challenge for agriculture. New solutions are required to solve this problem, and crop breeding for high nitrogen use efficiency or tolerance of low nitrogen availability has been widely considered to be a promising approach. However, the molecular mechanisms of high nitrogen use efficiency or low-nitrogen tolerance in crop plants are still to be elucidated, including the role of long non-coding RNAs (lncRNAs).

**Results:**

In this study, we identified 498 lncRNAs in barley (*Hordeum vulgare*) landrace B968 (Liuzhutouzidamai), of which 487 were novel, and characterised 56 that were responsive to low-nitrogen stress. For functional analysis of differentially-expressed lncRNAs, the gene ontology (GO) and Kyoto Encyclopedia of Genes and Genomes (KEGG) enrichment of co-expressed and co-located protein-coding genes were analyzed, and interactions with annotated co-expressed protein coding genes or micro RNAs (miRNAs) were further predicted. Target mimicry prediction between differentially-expressed lncRNAs and miRNAs identified 40 putative target mimics of lncRNAs and 58 target miRNAs. Six differentially-expressed lncRNAs were further validated by qPCR, and one in particular showed consistent differential expression using both techniques. Expression levels of most of the lncRNAs were found to be very low, and this may be the reason for the apparent inconsistency between RNA-seq and qPCR data.

**Conclusions:**

The analysis of lncRNAs that are differentially-expressed under low-nitrogen stress, as well as their co-expressed or co-located protein coding genes and target mimics, could elucidate complex and hitherto uncharacterised mechanisms involved in the adaptation to low-nitrogen stress in barley and other crop plants.

## Background

The remarkable changes and advances in crop production in the past half century have mainly been based on the incorporation of dwarfing genes into breeding programmes and the use of inorganic chemical fertilization: developments that are popularly defined as the Green Revolution [1, 2]. However, yield gains based on crop breeding and chemical fertilizer applications (especially nitrogen fertilizer) have reached a plateau [3] and excess utilization of chemical fertilizers causes serious environmental problems [4, 5]. On the other hand, the lack of chemical fertilizers is still a big problem in many developing countries and poor regions. Therefore, new solutions must be developed and adopted for increasing yields while maintaining or decreasing chemical nitrogen fertilizer applications. Consequently, high nitrogen use efficiency crop breeding, a concept that has existed for many decades, is becoming increasingly important.

Although the concept of nitrogen use efficiency was proposed as early as the 1980s [6], the availability and low cost of chemical fertilizer, together with the focus on dwarfing/semi-dwarfing genes in crop breeding, meant that there was little progress in improving this important trait [2]. Moreover, the molecular mechanisms of high nitrogen use efficiency were still not clear because of their complex nature [2, 7]. Presently, as chemical fertilizer becomes more expensive and its carbon footprint and environmental cost become increasingly unacceptable, more researchers are trying to address this and provide new solutions for improving nitrogen use efficiency of crops by conducting low-nitrogen tolerance studies [8–10].

Long non-coding RNAs (lncRNAs) are defined as RNAs that are longer than 200 nucleotides and have no protein coding potential. They can be classified into three types based on the positional relationship of the DNA regions that encode them with respect to protein coding genes: long intergenic non-coding RNAs (lincRNAs); antisense lncRNAs; and intronic lncRNAs (incRNAs) [11, 12]. LncRNAs received little attention for many years, but this changed after the discovery of X-inactive specific transcripts (XIST) in animal systems and the development of next generation sequencing (NGS) [11, 13]. LncRNAs are now thought to play important roles in transcriptional and post-transcriptional regulation, histone modification, RNA processing and small RNA pathways [13]. LncRNAs have also been investigated in many crops under different stresses, including nitrogen deficiency [10, 12, 14, 15], although there remains little information related to lncRNAs in barley, especially related to low-nitrogen tolerance.

Barley is the fourth largest cereal crop in the world and an important model plant for cereal research. It was one of the first domesticated cereal grains and has played an important role in humankind’s culture because of its suitability for malting [16]. Although crop selections based on preferences of farmers and breeders have had a big impact on modern crop cultivars [17], the lack of chemical fertilizer supply in barley landrace production in the past may mean that some germplasms with low-nitrogen tolerance have been retained, and these might be beneficial for improving nitrogen use efficiency if incorporated into breeding programmes. Thus, we made a screening of low-nitrogen tolerance in a barley landrace collection, and found that the restriction of growth was mainly in the shoots in the primary stage of the stress, while the landrace B968 (Liuzhutouzidamai) showed strong low-nitrogen tolerance [18–20]. Previously, we have shown changes in gene expression in barley shoots even at 1 h after low-nitrogen stress has been imposed [21], and the importance of gene regulation in shoots has also been observed in low-nitrogen stressed rice [22].

The aim of the present study was to conduct a comparative transcriptomic analysis to identify lncRNAs responsive to 1 h of low-nitrogen stress in shoots of barley landrace B968. These lncRNAs could potentially be exploited to improve low-nitrogen stress tolerance and/or nitrogen use efficiency in barley. Landrace B968 is considered to be relatively tolerant of low-nitrogen stress because its shoot biomass is unaffected by periods of low-nitrogen stress that cause reduced biomass in most barley landraces [19, 20]. Even so, shoot nitrogen (N) concentration (mg N per g dry weight of shoot) and N accumulation (mg N per plant) have both been shown to be significantly reduced under low-nitrogen stress, while root biomass is significantly increased. This suggests that the response to low-nitrogen stress is different in roots and shoots, and that shoot biomass more directly reflects the resistance to low-nitrogen stress, although the increase in root biomass may be beneficial for low-nitrogen adaptation [19–21].

The study is the first to investigate lncRNAs responsive to low-nitrogen stress in barley at the whole transcriptome level and to predict interactions between lncRNAs and protein coding genes or miRNAs.

## Results

### Identification and characterization of lncRNAs

Shoot samples of barley landrace B968 grown under normal nitrogen (N) supply and low-nitrogen stress conditions were used for cDNA library construction and RNA-seq analysis, with two biological replicates for each sample. The RNA-seq data have been deposited with the National Center for Biotechnology Information: Submission ID SUB6290350; BioProject ID PRJNA566107.

**Fig. 1:**
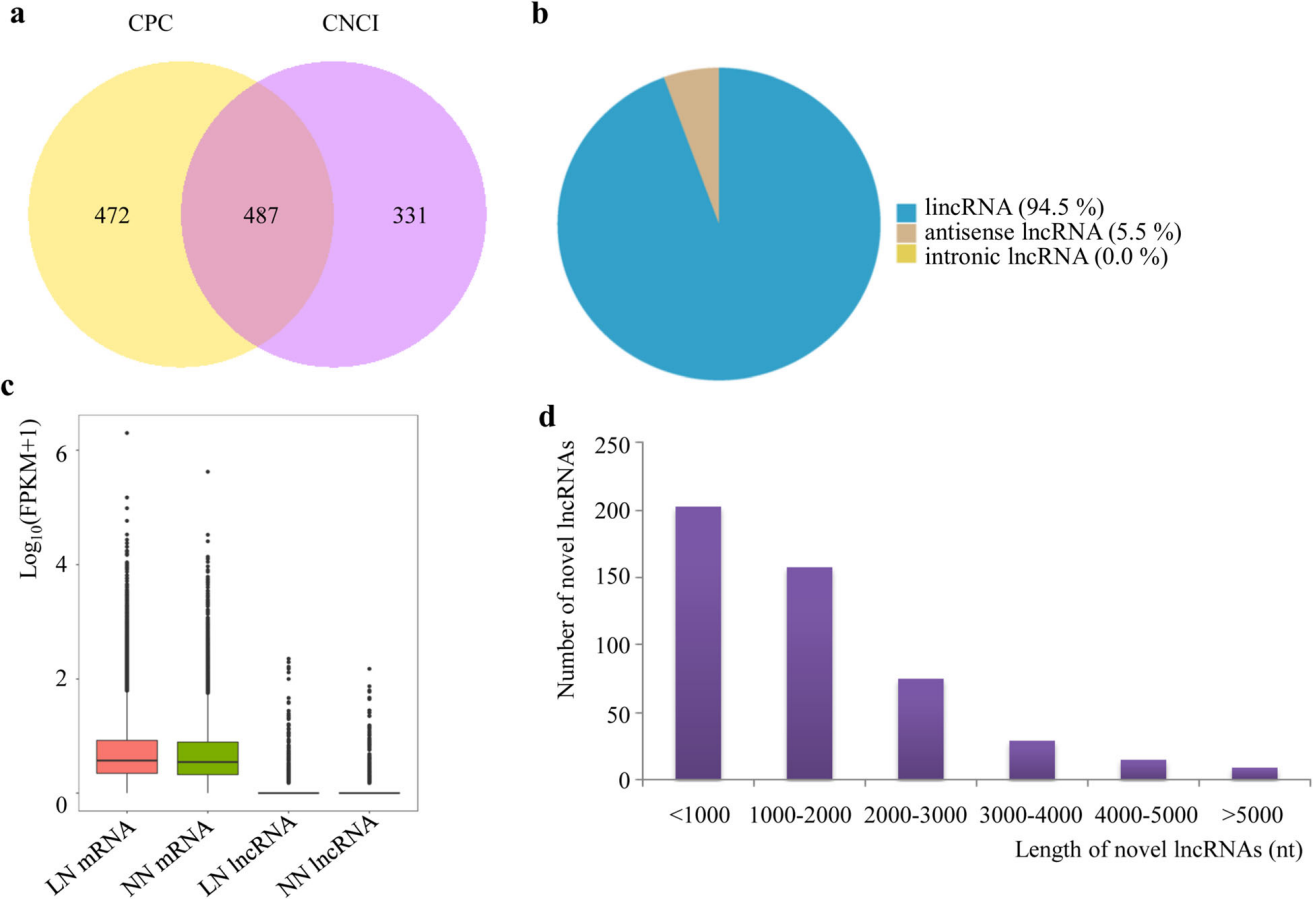
Identification and characterisation of novel lncRNAs expressed in shoots of barley under two different nitrogen treatments (normal nitrogen (NN) and low-nitrogen stress (LN)). **a** Venn diagram showing the numbers of novel lncRNAs identified by Coding Potential Calculator (CPC) and Coding Non-Coding Index (CNCI); **b** Classification of the lncRNAs identified in the study; **c** Overall expression levels Log_10_(fragments per kilobase of transcript per million mapped reads (FPKM) + 1) of lncRNAs and mRNAs in samples grown under the two nitrogen treatments; **d** Distribution of novel lncRNAs based on length

In total, 498 unique lncRNAs were identified in the RNA-seq data, of which 487 were novel, including 460 intergenic lncRNAs (lincRNAs) and 27 antisense lncRNAs (Fig. 1a and b, Additional file 2). They were located across all the chromosomes with the most abundant lncRNAs on Chromosome 2 and the least abundant on Chromosome 6, while they exhibited no preference for sense or antisense strand (Additional file 2). We also compared the expression levels of lncRNAs and mRNAs in the normal N and low N conditions and found that lncRNAs and mRNAs were each expressed at similar levels between the two treatments, while the overall expression level of lncRNAs was lower than that of mRNAs (Fig. 1c). We further analyzed the length of these novel lncRNAs and found that most of them were shorter than 2000 nt (Fig. 1d).

### Response of lncRNAs to low-nitrogen stress

**Fig. 2:**
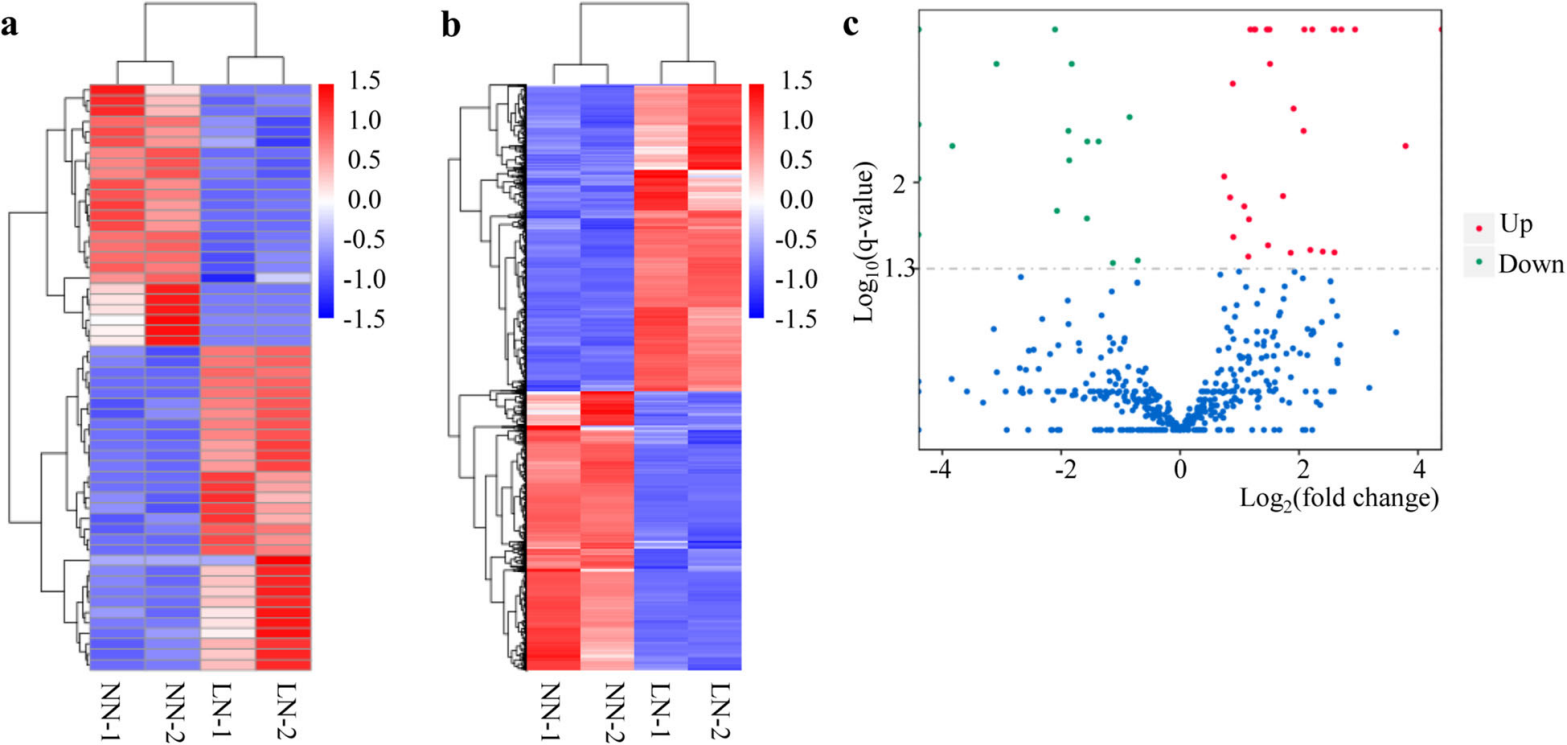
Analysis of transcripts in barley shoots grown under two nitrogen treatments, normal nitrogen (NN) and low-nitrogen stress (LN), with two replicates (1 and 2) for each. **a** Cluster analysis of specifically-expressed lncRNAs. **b** Cluster analysis of specifically-expressed mRNAs. **c** Differential expression of lncRNAs

To identify low-nitrogen stress responsive lncRNAs in shoots of barley, the normalized expression of lncRNAs was compared between the normal N and low N treatments. Clustering showed the lncRNAs to be grouped into two categories in the normal N and low N samples (Fig. 2a). The normalized expression of mRNAs was then compared between the normal N and low N treatments, and clustering also showed obvious differences between the normal N and low N samples for these (Fig. 2b). Fifty-six lncRNAs (all novel) showed differential expression between the normal N and low N treatments, of which 31 were up-regulated in the low N condition and 25 down-regulated (Fig. 2c, Additional file 2). The low-nitrogen stress responsive lncRNAs were found to be located across all of the chromosomes with the most abundant lncRNAs on Chromosomes 2 and 6. Chromosome 6 had the highest ratio of low-nitrogen responsive lncRNAs (Additional file 2).

### Functional analysis of low-nitrogen stress responsive lncRNAs

**Fig. 3:**
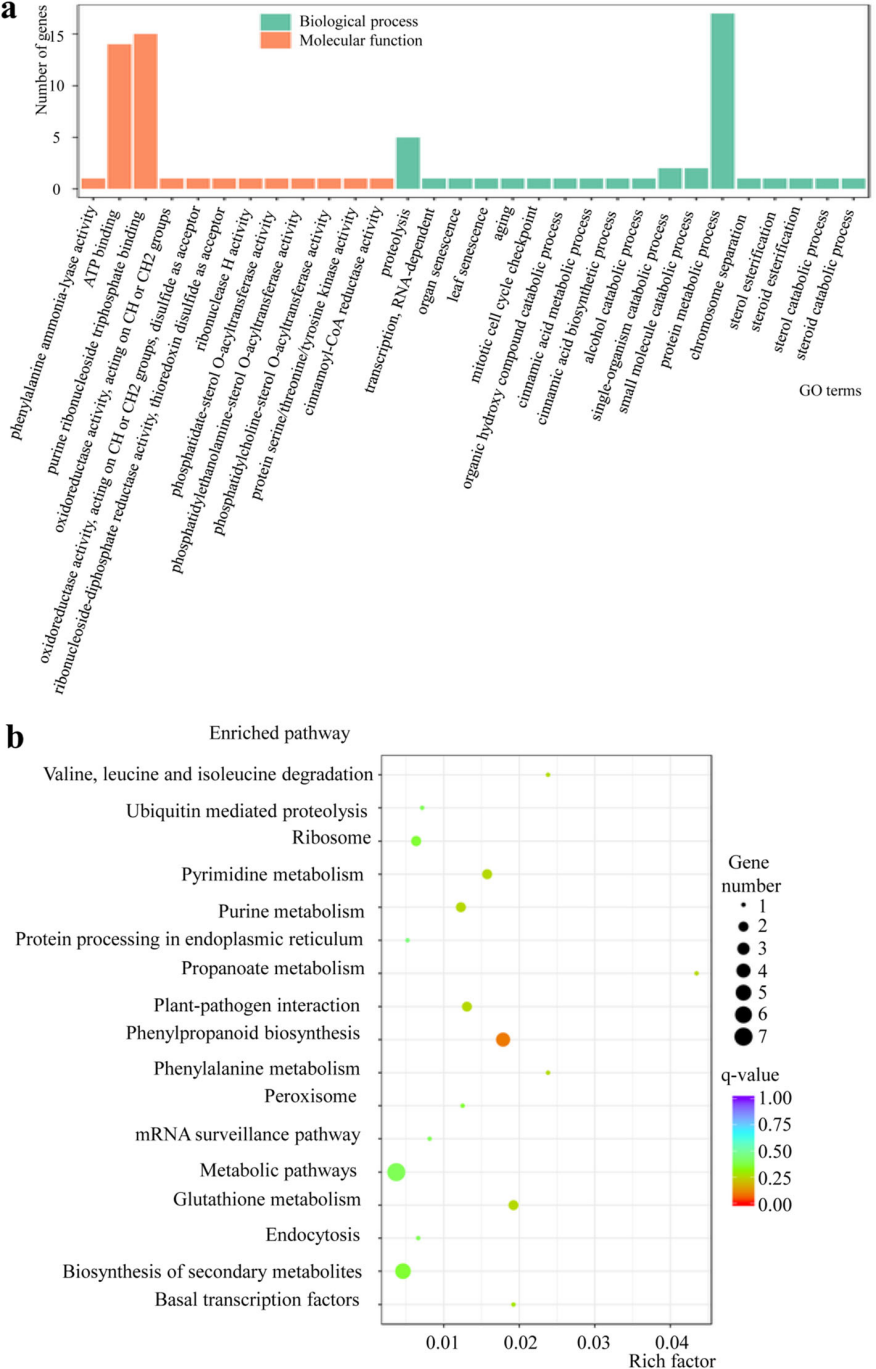
**a** GO and **b** KEGG analysis of protein coding genes co-located with lncRNAs that are differentially-expressed in response to low-nitrogen stress in barley shoots

To analyze the potential functions of the low-nitrogen stress responsive lncRNAs, co-located target genes, i.e. genes that were located in the genome less than 100 kb away from the differentially-expressed lncRNA region, were used for GO and KEGG analysis (Additional file 2). The GO analysis of these co-located genes showed that the top 30 terms were mainly enriched in categories of biological process and molecular function, especially the three terms of ATP binding, purine ribonucleoside triphosphate binding and protein metabolic process, although this was not significant (Fig. 3a and Additional file 2). The KEGG enrichment showed that these co-located genes were assigned to 17 KEGG pathways, but only the phenylpropanoid biosynthesis pathway was significant (*p*-adj < 0.05) (Fig. 3b and Additional file 2).

**Fig. 4:**
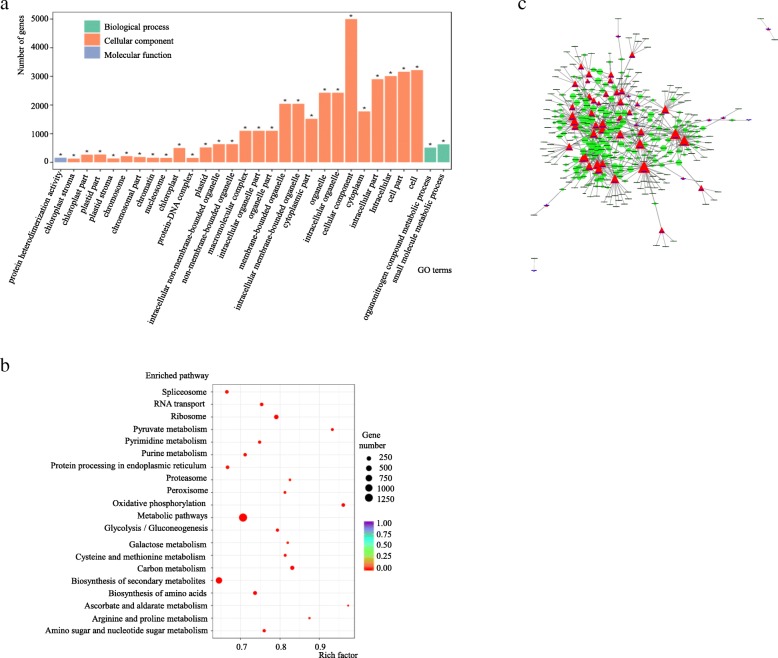
Functional analysis of protein coding genes co-expressed with lncRNAs that are differentially-expressed in response to low-nitrogen stress in barley shoots. **a** GO analysis; **b** KEGG analysis; **c** Cytoscape network of differentially-expressed lncRNAs and co-expressed protein coding genes: red triangles represent lncRNAs and green circles represent protein coding genes, with the size and complexity of the network reflecting the number of interactions involved (Additional file 2)

Co-expressed protein coding genes (i.e. genes for which co-expression with a lncRNA showed a Pearson correlation coefficient above 0.95 and *p* value less than 0.001) were used for GO and KEGG analysis (Additional file 2). The GO analysis of these co-expressed protein coding genes indicated that the top 30 terms were mainly enriched in the category of cellular component, with all 30 terms significant (*p*-adj < 0.05), and the category of cellular component was the largest (Fig. 4a and Additional file 2). The KEGG analysis indicated that the top 20 enriched pathways were all significant (*p*-adj < 0.05). The three categories of metabolic pathway, biosynthesis of secondary metabolites and ribosome were the largest, while pyruvate metabolism, oxidative phosphorylation and ascorbate and aldarate metabolism had the highest enrichment factors (Fig. 4b and Additional file 2).

To elucidate the function of differentially-expressed lncRNAs under low-nitrogen stress and the relationship between lncRNAs and protein coding genes, annotated co-expressed protein coding genes and differentially-expressed lncRNAs were further used to establish putative interaction networks by using Cytoscape (Fig. 4c and Additional file 2). The analysis indicated that the interaction between protein coding genes and lncRNAs was very complicated, and one lncRNA could interact with more than one protein coding gene and one protein coding gene could also interact with more than one lncRNA. Clearly, those lncRNAs that can interact with many protein coding genes, such as lnc000327, could potentially have great effects on the response to low-nitrogen stress.

### Target mimicry of lncRNAs to m iRNAs

**Fig. 5:**
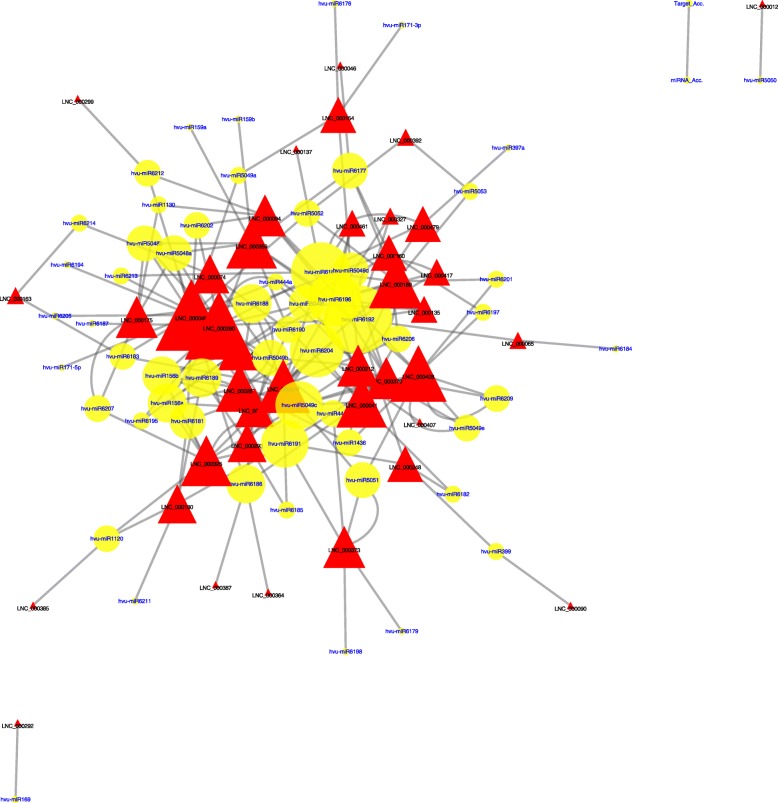
Cytoscape network of differentially-expressed lncRNAs and miRNAs. The red triangles represent lncRNAs and the yellow circles miRNAs, with the size and complexity of the network reflecting the number of interactions involved (Additional file 2)

Forty putative target mimics of lncRNAs and 58 target miRNAs were identified in total, including one lncRNA that could interact with more than one miRNA, and one miRNA that could interact with two or more different lncRNAs (Fig. 5 and Additional file 2). Although the action of lncRNAs to miRNAs needed to be further validated, the prediction still provided an overview of the potential mechanism of target mimicry.

### Validation of differentially-expressed lncRNAs by qPCR

**Fig. 6:**
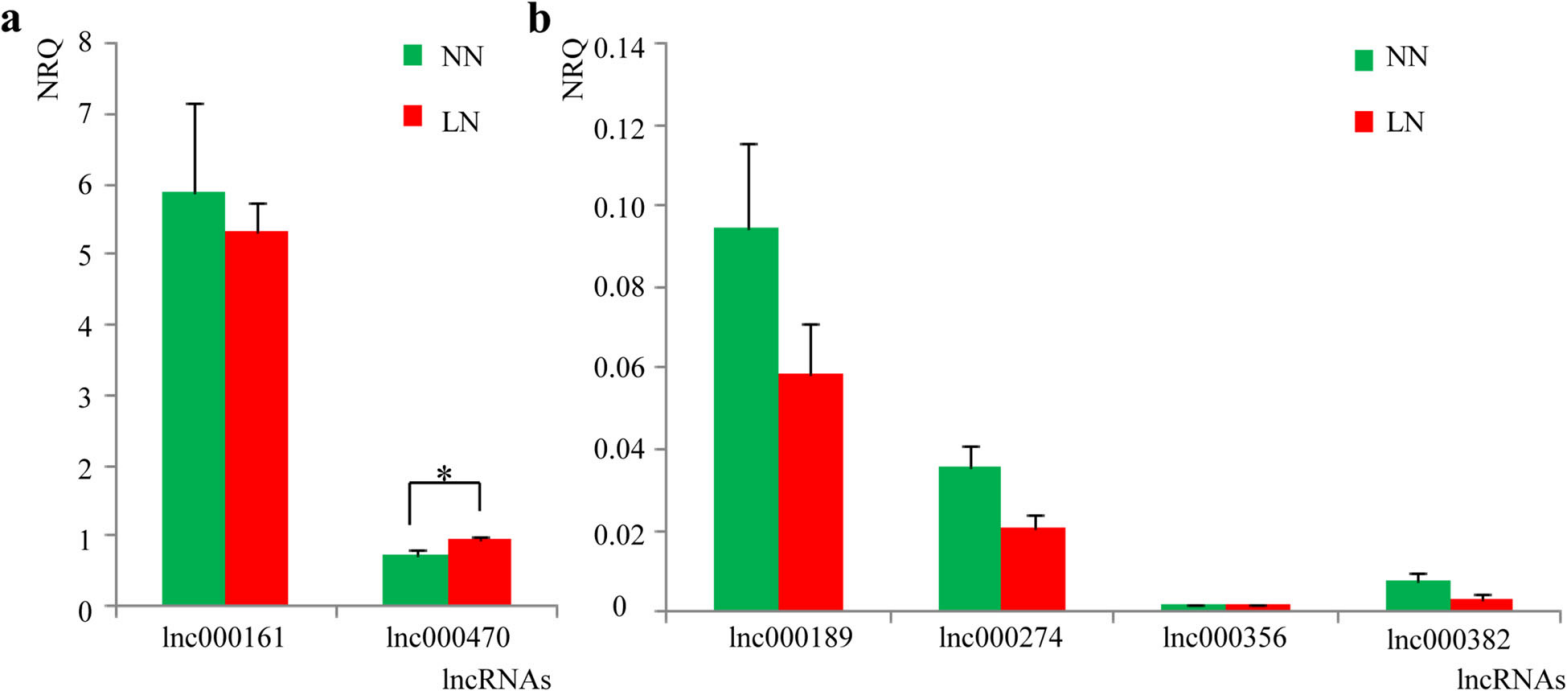
Relative gene expression (normalized relative quantity (NRQ)) of lncRNAs from barley shoots growing under normal nitrogen (NN) and low-nitrogen stress (LN) conditions, analysed by qPCR. Means and standard errors are shown, and * indicates significantly differential expression (*p* < 0.05, t-test) between the treatments. a. lnc000161 and lnc000470; b. lnc000189, lnc000274, lnc000356 and lnc000382

Differentially-expressed lncRNAs with read numbers above 30 in either the normal N or low N condition were selected for qPCR validation. These were: lnc000161, lnc000189, lnc000274, lnc000356, lnc000382, lnc000470 and lnc000182, although lnc000182 was dropped because its primer design proved very difficult. Of the others, only lnc000470 showed consistency with the RNA-seq results, being up-regulated in the low N condition (Fig. 6a and b). The expression levels of the lncRNAs were much lower than for the reference genes, consistent with the expectation for lncRNAs (Fig. 1c), with the exception of lnc000161, which was relatively highly expressed in both treatments (Fig. 6a). The low expression of the lncRNAs may be the cause of the apparent discrepancy between the qPCR and RNA-seq results, and alternatives to qPCR may need to be developed to validate the expression of lncRNAs. Nevertheless, lnc000470, for which the qPCR result did validate the RNA-seq data, along with its interacting protein coding genes and miRNAs, is a good candidate for use in further studies.

## Discussion

Improving nitrogen use efficiency at very high nitrogen conditions might be very difficult or even impossible [2], so mechanisms focused on improving resistance to low-nitrogen or nitrogen starvation stresses are thought to be better options for improving nitrogen use efficiency as well as adaptation to poor lands. A successful example is the discovery of the early nodulin gene in rice, which was identified by transcriptional analysis under low-nitrogen stress conditions and is associated with improved nitrogen use efficiency [8]. The discovery of this gene and its association with improved resistance to low-nitrogen stress and better nitrogen use efficiency is clear evidence that related studies should not be restricted to genes directly involved in nitrogen metabolism. Meanwhile, barley, with its good adaptation to poor lands, is becoming the model crop of choice for similar studies, especially with the recent development of new sequencing technologies [9, 23, 24].

In this study, we firstly identified 487 novel barley lncRNAs at the transcriptome level, most of which comprised less than 2000 nucleotides, much like the situation in cotton [14]. Moreover, 56 lncRNAs were differentially-expressed in landrace B968 under low-nitrogen stress and, therefore, might play important roles in the response to low-nitrogen stress, of which 55 were classified as lincRNAs. For functional analysis of lncRNAs responsive to low-nitrogen stress, we focused mainly on their relationship with protein coding genes and miRNAs. For co-located protein coding genes, there was no significantly enriched term in GO analysis, and only one pathway, phenylpropanoid biosynthesis, that was significantly enriched. Notably, the phenylpropanoid biosynthesis pathway has also been shown to be enriched in Tibetan wild barley in response to low nitrogen [9]. This suggests that genes related to phenylpropanoid biosynthesis might be important in the response to low-nitrogen stress in barley. For this analysis, we set a separation distance limit of 100 kb, which might explain why relatively few genes were identified and only one pathway was shown to be enriched.

In contrast, the analysis of co-expressed protein coding genes identified huge numbers of genes, and the GO analysis showed that all of the top 30 enriched terms were significant, with most belonging to the category of cellular component. The KEGG analysis also showed that all top 20 enriched pathways were significant. It indicated that the functional terms or pathways were broadly associated with the response to low-nitrogen stress, consistent with previous studies [9, 22, 23, 25]. To give a more intuitive description of the relationship between differentially-expressed lncRNAs and co-expressed protein coding genes, the cytoscape network clearly identified the hub differentially-expressed lncRNAs, and this network also could help us to investigate different hub lncRNAs, together with their targeted protein coding genes, for resistance to low-nitrogen stress.

Target mimicry was firstly proposed in Arabidopsis, and a lncRNA called *IPS1* was identified that could bind to a miRNA (miR399) to prevent the degradation of its target mRNA, *PHO2*, to control Pi homeostasis [26]. This important function of lncRNAs is unlikely to be limited to phosphate starvation [10, 13, 27]. Forty putative target mimics of lncRNAs identified in this study may be used for investigation of their regulation of miRNAs and their target mRNAs to reveal more complicated mechanisms for adaptation to low-nitrogen stress. Moreover, we also found that lnc00090 and lnc000248 were target mimics for hvu-miR399, suggesting that miR399 might also play important roles under low-nitrogen stress in barley.

## Conclusions

The study shows that the analysis of lncRNAs that are differentially-expressed under low-nitrogen stress, as well as their co-expressed or co-located protein coding genes and target mimics, could elucidate complex and hitherto uncharacterised mechanisms involved in the adaptation to low-nitrogen stress in barley and other crop plants.

## Methods

### Plant materials and low nitrogen treatments

Barley landrace B968 was one of a collection of barley genotypes originally obtained as seed from the Shanghai Agrobiological Gene Center, Shanghai, China, and maintained at the Biotechnology Research Institute of Shanghai Academy of Agricultural Sciences by Zhiwei Chen and Qi Jiang. Plants of landrace B968 were grown in an artificial climate chamber, with growth conditions as described by Chen et al. [21]. NH_4_NO_3_ was used as nitrogen source, and there were two nitrogen conditions: Normal nitrogen (N) (control), with 1.43 mM NH_4_NO_3_, and low-nitrogen stress, with 0.24 mM NH_4_NO_3_. Low-nitrogen treatment was applied from the 3-leaf stage of seedling development (before this stage, we consider that the seed endosperm could still be providing nitrogen to the growing seedling). Shoots for transcriptome analysis and lncRNA expression validation were sampled after 1 h of treatment in both nitrogen conditions and kept in a − 80 °C freezer. There were two replicates of each sample for transcriptome analysis and three replicates of each sample for qPCR analysis. For traits investigation, seedlings were harvested after 2 weeks of treatments under the two nitrogen conditions, according to Chen et al. [20, 21].

### Library preparation and RNA-sequencing for lncRNA

Total RNA was isolated from each barley shoot sample using TRIzol reagent (Invitrogen, USA). RNA degradation and contamination was monitored by electrophoresis using 1% agarose gels; purity was checked using the NanoPhotometer spectrophotometer (IMPLEN, CA, USA); concentration was measured using a Qubit RNA Assay Kit and Qubit 2.0 Flurometer (Life Technologies, CA, USA); and integrity was assessed using the RNA Nano 6000 Assay Kit of the Bioanalyzer 2100 system (Agilent Technologies, CA, USA). A total amount of 3 μg total RNA per sample was used as input material for the RNA sample preparations, and ribosomal RNA was removed using an Epicentre Ribo-zero rRNA Removal Kit (Epicentre, Madison, WI, USA). Sequencing libraries were then generated using a NEBNext® Ultra™ Directional RNA Library Prep Kit for Illumina (NEB, USA), according to the manufacturer’s instructions, using the rRNA-depleted RNA. The libraries were sequenced on an Illumina Hiseq Xten platform (Illumina Inc., San Diego, CA) and 150 bp paired-end reads were generated. The clean nucleotide sequence data ranged from 16.14 to 19.64 Gb (all > 12 Gb), and the Q30 percentages were all > 95% (Table 1, Additional file 1, Fig. A1). These results indicated that the data were sufficient and reliable enough for further analysis. Spearman correlation analysis also showed that the two biological replicates of each sample met the requirements (all over 0.8) (Additional file 1, Fig. A2).

**Table 1:** Summary of RNA-seq data from barley roots grown under normal nitrogen (NN) and low nitrogen (LN) conditions, with two biological replicates for each treatment

	NN		LN	
	1	2	1	2
Raw bases (Gb)	18.77	19.20	16.33	19.88
Clean bases (Gb)	18.36	18.81	16.14	19.64
Clean Q30 (%)	95.17	95.04	95.75	95.45
Clean GC	43.89	43.73	46.24	46.55

### Novel lncRNA identification

Clean data were obtained by removing reads containing adapter sequences or poly-N, and other low-quality reads from the raw data. Q20, Q30 and GC information were calculated to evaluate the clean data. Reference genome and gene model annotation files were downloaded from EnsemblPlants (http://plants.ensembl.org/Hordeum_vulg are/Info/Index, IBSC v2). Index of the reference genome was built using Bowtie v2 and clean reads were aligned to the reference genome using TopHat v2.0.9. The mapped reads of each sample were assembled by Cufflinks (v2.1.1) in a reference annotation-based transcripts (BRAT) method [28, 29]. Six steps were adopted to identify novel lncRNAs: 1) transcripts with exon count ≥2 were selected; 2) transcripts with length > 200 bp were selected; 3) transcripts with a coverage of > 3 calculated by cufflinks were selected; 4) transcripts of known mRNAs (protein-coding) or ncRNAs were removed through Cuffcompare; 5) transcripts with expression of fragments per kilobase of transcript per million mapped reads (FPKM) ≥ 0.5 were selected; 6) transcripts with non-coding potential were detected by both CPC (Coding Potential Calculator) (0.9 - r2) [30] and CNCI (Coding Non-Coding Index) (v2) [31].

### Differential expression of lncRNAs

Cuffdiff v2.1.1 was used to provide statistical routines for determining differential expression in digital transcript or gene expression data using a model based on the negative binomial distribution [28]. Transcripts with a *p*-adj value (adjusted *p* value) < 0.05 were assigned as significantly differentially-expressed.

### GO and KEGG analysis

In order to predict the function of low-nitrogen stress responsive lncRNAs, gene ontology (GO) enrichment analysis of co-located or co-expressed protein coding genes by differentially-expressed lncRNAs were respectively implemented by the GOseq R package, and GO terms with a *p*-adj value less than 0.05 were considered significantly enriched. Kyoto Encyclopedia of Genes and Genomes (KEGG) (http://www.genome.jp/kegg/) pathways enrichment analysis was conducted using KOBAS software.

### Co-expressed protein coding genes and lncRNA interaction

Annotated co-expressed protein coding genes with Pearson correlation coefficient above 0.99 and *p* value less than 0.001 and differentially-expressed lncRNAs were used for putative interactive network prediction by using Cytoscape.

### M iRNA and lncRNA interaction

All barley miRNAs were downloaded from Mirbase 22 (http://www.mirbase.org/) and all significantly differentially-expressed lncRNAs were used for target mimicry prediction by psRNATarget. The criteria and principles for prediction target mimics were mainly based on Deng et al. [14] and Wu et al. [27]. Differentially-expressed lncRNAs and their target miRNAs were used for putative interactive network prediction using Cytoscape.

### Validation by quantitative real-time PCR and statistics

Total RNA was isolated using TRIzol reagent (Invitrogen, USA) and treated with RNase-free DNase I (Promega, USA). PrimeScript® RT reagent Kit (TaKaRa, Japan) was used to reverse-transcribe approximately 1 μg RNA into first-strand cDNA. Quantitative real-time PCR (qRT-PCR) was performed using SYBR® Select Master Mix and an ABI 7500 Fast Instrument (Applied Biosystems, USA). All qRT-PCR reactions were performed in triplicate for each cDNA sample with an annealing temperature of 60 °C and a total of 40 cycles of amplification, and each reaction contained 5 μL 2 × mix, 0.6 μL of each primer (10 mM) and 1 μL 10 × diluted cDNA template in a final volume of 10 μL. Primers used for lncRNAs and reference genes are listed in Additional file 2. Those for lncRNAs were designed by Primer-BLAST on the NCBI website, while primers for reference genes were directly taken from Chen et al. [24]; PCR efficiencies were accessed by LinRegPCR software. The normalized relative quantity of each lncRNA was calculated according to Chen et al. [32] and Rieu and Powers [33], and Cq values were also obtained by LinRegPCR software. Three reference genes were used for the calculation: *HvGAPDH* (glyceraldehyde-3-phosphate dehydrogenase), *HvARF1* (ADP-ribosylation factor 1-like) and *HvTUBB6* (beta tubulin 6). These were the three most stable reference genes identified in shoots of barley under low-nitrogen stress [24]. Statistical analysis was mainly according to Chen et al. [32] using transformed Cq values [Cq = log_2_ (1/NRQ)]. The statistical significance of differences in gene expression between the control and low-nitrogen stress was evaluated by t-test at 0.05 level (*p* < 0.05) (Additional file 1).

## Supplementary information

**Additional file 1.** : Figures A1 and A2.

**Additional file 2.** lncRNAs identified in the study, Nucleotide sequences of lncRNAs identified in the study, Differentially-expressed lncRNAs, KEGG analysis of co-located protein coding genes, GO analysis of co-located protein coding genes, KEGG analysis of co-expressed protein coding genes, GO analysis of co-expressed protein coding genes, Cytoscape analysis: Annotated co-expressed protein coding genes by differentially-expressed lncRNAs, Putative target mimics of lncRNAs for miRNAs, Primers used in the study.

## Data Availability

The RNA-seq data have been deposited with the National Center for Biotechnology Information: Submission ID SUB6290350; BioProject ID PRJNA566107.

## Abbreviations

CNCI: Coding non-coding index
CPC: Coding potential calculator
Cq: Quantitation cycle
FPKM: Fragments per kilobase of transcript per million mapped reads
GO: Gene ontology
KEGG: Kyoto Encyclopedia of Genes and Genomes
lncRNA: Long non-coding RNA
lincRNA: Long intergenic non-coding RNA
incRNA: Intronic non-coding RNA
miRNA: Micro RNA
NGS: Next generation sequencing;
qPCR: Quantitative polymerase chain reaction
p-adj: Adjusted *p* value
RNA-seq: RNA sequencing
XIST: X-inactive specific transcripts
