# Supplementary material for: Novel low-nitrogen stress-responsive long non-coding RNAs (lncRNA) in barley landrace B968 (Liuzhutouzidamai) at seedling stage
Source: BMC Plant Biol. 2020 Apr 6;20:142. doi: 10.1186/s12870-020-02350-2 (PMC7137197; doi:10.1186/s12870-020-02350-2)

**Novel low nitrogen stress-responsive long non-coding RNAs (lncRNA)** **in barley at seedling stage**

**Zhiwei Chen****^1,2,3*^, Qi Jiang^1,3*^, Panpan Jiang^4^, Wan Zhang^5^, Jianhua Hu ang^1,2^, Chenghong Liu^1,2†^, Nigel G. Halford^6†^, Ruiju Lu^1,2†^**

***1. Biotechnology Research Institute, Shanghai Academy of Agricultural Sciences, Shanghai 201106, China***

***2. Shanghai Key Laboratory of Agricultural Genetics and Breeding, Shanghai 201106, China***

***3. College of Fisheries and Life Science, Shanghai Ocean University, Shanghai 201306, China***

***4. Shenzhen RealOm ics (Biotech) Co., Ltd., Shenzhen 518081, China***

***5. Suzhou Polytechnic Institute of Agriculture, Suzhou, Jiangsu 215008, China***

***6. Plant Sciences Department, Rothamsted Research, Harpenden, Hertfordshire AL5 2JQ, United Kingdom***

^*^Co-first authors. ^†^Corresponding authors: Telephone 86-021-62203071, email [Chliu001@saas.sh.cn](mailto:Chliu001@saas.sh.cn) (CL); Telephone: 44 1582 938203, email [nigel.halford@rothamsted.ac.uk](mailto:nigel.halford@rothamsted.ac.uk) (NGH); Telephone 86-021-62202965, email [luruiju62@163.com](mailto:luruiju62@163.com) (RL).

**ADDITIONAL FILE 1**

**Figure A1**

RNA-seq quality control: Quality score distribution along reads in four samples. A. NN-1. B. NN-2. C. LN-1. D. LN-2. NN-1,2 represent two biological replicates of sample under normal nitrogen (NN) supply (the control); LN-1,2 represent two biological replicates of sample under low nitrogen (LN) stress.

**
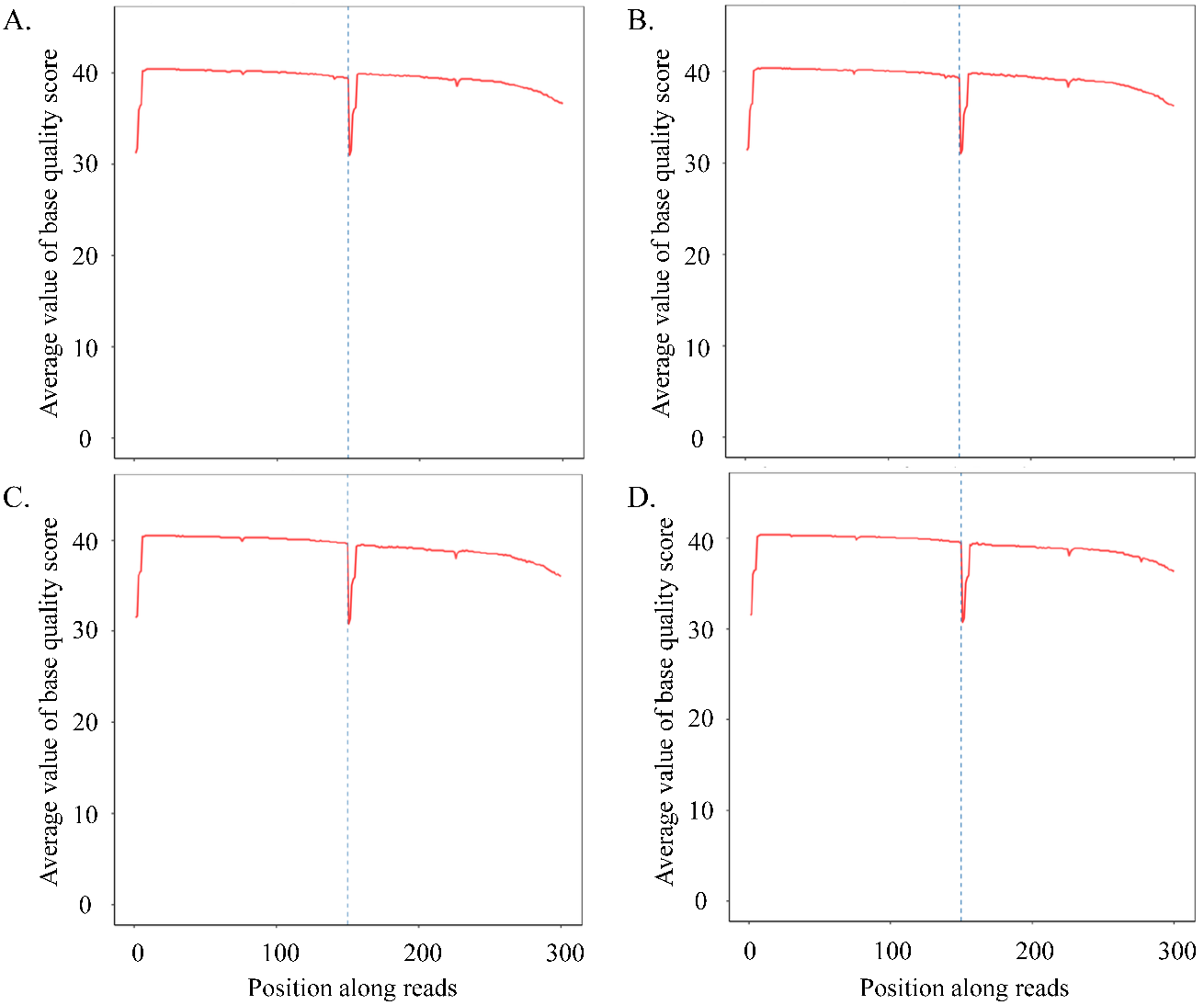
**

**Figure A2**

RNA-seq quality control: Spearman correlations comparing all samples, including two biological replicates of each treatment. NN-1,2 represent two biological replicates of shoots growing under normal nitrogen (NN); LN-1,2 represent two biological replicates of shoots growing under low nitrogen (LN) stress.


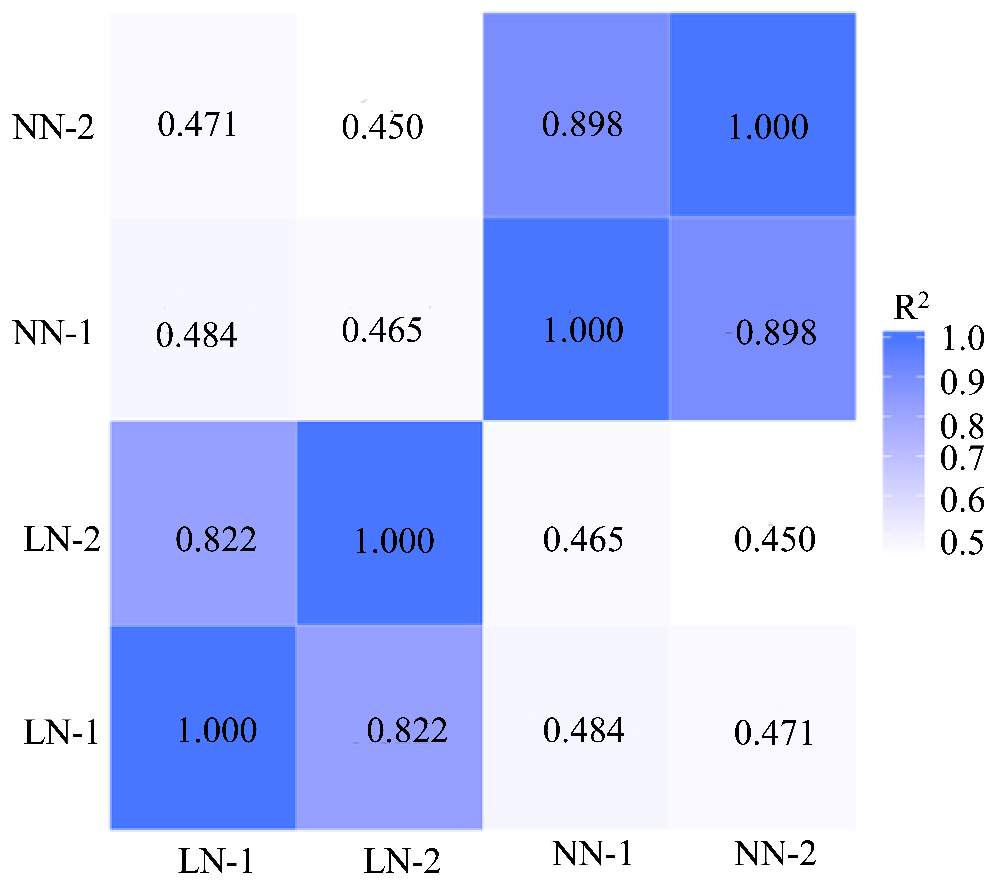

Supplement: Supplementary file 1 — Additional file 1. : Figures A1 and A2. [file 12870_2020_2350_MOESM1_ESM.docx]
